# Utilizing Big Data to Identify Tiny Toxic Components: *Digitalis*

**DOI:** 10.3390/foods10081794

**Published:** 2021-08-03

**Authors:** Elizabeth Sage Hunter, Robert Literman, Sara M. Handy

**Affiliations:** Center for Food Safety and Applied Nutrition, Office of Regulatory Science, U.S. Food and Drug Administration, College Park, MD 20740, USA; Elizabeth.Hunter@fda.hhs.gov (E.S.H.); Robert.Literman@fda.hhs.gov (R.L.)

**Keywords:** dietary supplements, genome skimming, *Digitalis*, toxic botanicals

## Abstract

The botanical genus *Digitalis* is equal parts colorful, toxic, and medicinal, and its bioactive compounds have a long history of therapeutic use. However, with an extremely narrow therapeutic range, even trace amounts of *Digitalis* can cause adverse effects. Using chemical methods, the United States Food and Drug Administration traced a 1997 case of *Digitalis* toxicity to a shipment of *Plantago* (a common ingredient in dietary supplements marketed to improve digestion) contaminated with *Digitalis lanata*. With increased accessibility to next generation sequencing technology, here we ask whether this case could have been cracked rapidly using shallow genome sequencing strategies (e.g., genome skims). Using a modified implementation of the Site Identification from Short Read Sequences (SISRS) bioinformatics pipeline with whole-genome sequence data, we generated over 2 M genus-level single nucleotide polymorphisms in addition to species-informative single nucleotide polymorphisms. We simulated dietary supplement contamination by spiking low quantities (0–10%) of *Digitalis* whole-genome sequence data into a background of commonly used ingredients in products marketed for “digestive cleansing” and reliably detected Digitalis at the genus level while also discriminating between *Digitalis* species. This work serves as a roadmap for the development of novel DNA-based assays to quickly and reliably detect the presence of toxic species such as *Digitalis* in food products or dietary supplements using genomic methods and highlights the power of harnessing the entire genome to identify botanical species.

## 1. Introduction

In 1785, physician and amateur botanist William Withering introduced the medical world to *Digitalis purpurea.* While he successfully used *Digitalis* preparations to treat a broad variety of cardiac conditions related to fluid retention, withering himself also noted the toxicity of this plant alongside its medicinal value, cautioning its use be discontinued as soon as symptoms of toxicity arose [1]. At the height of its use in the 1960s and 1970s, surveys estimated that up to 15% of all hospital patients were using digitalis-derived medications, and 20–30% of those patients were likely experiencing symptoms of toxicity [2]. These symptoms include cardiac manifestations, vomiting, anorexia, diarrhea, dizziness, fatigue, delirium, syncope, and visual disturbances such an xanthopsia (yellow vision) [1,3,4], and it has even been suggested that Vincent Van Gogh was experiencing xanthopsia due to digitalis toxicity during the last years of his life [5]. Today, two cardiac glycosides are still isolated for use from *Digitalis*: digitoxin and digoxin, and Withering’s warning regarding their narrow therapeutic range holds true almost 250 years later. Due to the risks of toxicity, *Digitalis* derived drugs have become less popular in modern medicine [6], but it’s potent toxic effects still pose risks in cases of accidental contamination.

In 1997, two women were hospitalized with symptoms consistent with *Digitalis* toxicity several days after starting a dietary supplement regiment intended to provide “internal cleansing” [7]. The cases were brought to the United States Food and Drug Administration (FDA), which investigated them using a chemical-based method including a Kedde reaction and thin layer chromatography. The presence of cardiac glycosides was confirmed in the raw materials indicated by the initial analyses using liquid chromatography and mass spectroscopy, and the species (*Digitalis lanata*) was confirmed using microscopy [7]. The source was identified as a batch of *Plantago,* which is commonly known as plantain, although not related to the tropical banana plant [7].

The world of eukaryotic DNA-based species identification in food and dietary supplements products has undergone a revolution in the past 10 years, and while progress has been swift it has not been without challenges [8,9,10]. Contemporary methods of DNA-based identification include DNA fingerprinting techniques like restriction fragment length polymorphisms [11], microarrays [12], a full suite of polymerase chain reaction (PCR) methods, [13,14,15,16,17], in addition to traditional DNA barcoding [9,18] and a variety of next generation sequencing (NGS) approaches [19,20,21].

NGS methods have recently been investigated to distinguish between closely related eukaryotic species [21,22] and to detect food origins [23], the presence of toxic species [24], allergens [25], as well as other cases of adulteration [20,26,27], both accidental and economically motivated. However, the application of NGS technology is not without limitations. Targeted barcoding regions can suffer from amplification and primer bias, while whole-genome sequencing (WGS) methods are often restricted by taxonomically limited sequence databases and lack of well-annotated reference genomes, especially for non-model organisms. Chloroplast databases can provide a uniform and informative reference for identification, but certain closely related or hybridized species are indistinguishable or confounded using these data [25]. Additionally, although chloroplast DNA is typically found in high copy numbers in many plant cells, plastid DNA proportions have been shown to vary due to both tissue type [28] and plant age [29], and large nuclear genomes can decrease the relative representation of plastid DNA in a genome skimming run [30,31,32].

Nuclear DNA methods may be required for robust detection of trace adulteration, but quality whole-genome references for flowering plants are still few and far between due to their size and repetitive structure. As of 2017, only 236 angiosperm genomes had been sequenced [33] out of an estimated 350,699 species (www.theplantlist.org, accessed on 17 May 2021). This represents less than 0.07% of angiosperms, and a curated but comprehensive database remains an elusive goal. Data from the broadest available nuclear databases (e.g., NCBI) can suffer from poor sequence quality, inaccurate identification, and contamination [34]. Voucher collections have been leveraged to mediate some of these problems by providing traceable, expertly identified reference material for chemical analyses [35]. At the FDA, these samples are also being used to facilitate the creation of DNA-based reference databases [36] and novel molecular pipelines [25] that could circumvent complications involved with chemical analyses [10].

In this study we present a modified implementation of Site Identification from Short Read Sequences (SISRS) [37], a reference-free ortholog discovery pipeline that takes WGS data as input, to identify toxic adulterants (*Digitalis*) in mixed samples both at the genus and species level. SISRS does not rely on external databases or annotated reference genomes (which do not exist for *Digitalis*) when identifying loci useful for classification, overcoming a major hurdle in many contemporary NGS pipelines [34]. From these SISRS loci we identify over 2 million genus-informative single nucleotide polymorphisms (SNPs) for *Digitalis* and thousands of species-informative SNPs. As in the real-world case of *Digitalis* toxicity, we spike *Digitalis* WGS data into simulated mixtures containing *Plantago*, another member of the Plantaginaceae family, along with other commonly used botanical ingredients in products marketed for digestive health. SNPs generated in this study facilitated robust detection of *Digitalis* adulteration at read proportions of 0.05%, and accurate species identification when datasets contained ~4 Mb of *Digitalis* data. Using test species that allow us to assess the sensitivity and specificity of our markers even when differentiating amid closely related botanicals, here we examine how methods utilizing the nuclear data from low-coverage WGS datasets (i.e., ‘genome skims’) might be used in the future to detect even minute amounts of contamination with toxic plant species in foods and dietary supplements.

## 2. Materials and Methods

### 2.1. Sample Acquisition

#### 2.1.1. DNA Extraction and Sequencing

DNA was extracted from thirty-two vouchered Plantaginaceae specimens from the New York Botanical Gardens (https://www.nybg.org/plant-research-and-conservation/tour/laboratory-collections accessed on 17 May 2021) and the University of Rhode Island’s Heber W. Youngken Medicinal Garden (https://web.uri.edu/youngken-garden/ accessed on 17 May 2021) using the QIAGEN DNeasy Plant Mini Kit. Samples were leaf material dried on silica, which were held at 4 C until extraction. The samples included species from the Plantaginaceae family: three *Digitalis,* eleven *Plantago,* fifteen *Veronica,* and three additional species (*Callitriche stagnalis*, *Bacopa monneri*, and *Gratiola ramosa*). Samples were quantified with a broad range Qubit 3 fluorometer assay to ensure successful extraction. Libraries were then prepared for Illumina sequencing, following [38], using a Covaris M220 sonicator to shear to approximately 300 bp, followed by end repair, adapter ligation, amplification, and subsequent AmPure cleanups using the KAPA HyperPrep kit and dual indexed adapters. Libraries were quantified and sized using a Qubit 3 fluorometer and an Agilent 2100 Bioanalyzer, respectively. Nine samples underwent an additional AmPure bead cleanup and quality control process to remove residual primer dimers. Samples were pooled at a 6nM concentration and sequenced on a single lane of the Illumina HiSeq2500 platform (Genewiz, South Plainfield, NJ, USA). The same libraries of *Digitalis grandiflora*, *D. ferruginea*, *D. lutea*, and also *Plantago patagonica* and *P. rhodosperma* were also sequenced on an in-house Illumina MiSeq with a 600-cycle kit, along with an additional independent sample of *D. ferruginea*. Five *Digitalis* samples were additionally sequenced on an in-house Illumina NextSeq500, including the three *Digitalis* libraries from the original HiSeq2500 run, and two additional vouchered samples provided by the University of Mississippi. These last two samples were extracted and prepared for sequencing using the same methods as above. Raw reads from these sequencing runs were deposited under the BioProject PRJNA325670, and their SRA accessions and associated BioSample IDs are available in the Supplementary Tables S2 and S5).

#### 2.1.2. Companion Data from Public Archives

In addition to specimens sequenced as part of this study, we also acquired publicly available sequence data for an additional *Digitalis purpurea* sample, as well as several Plantaginaceae species (*Littorella uniflora*, *Plantago lagopus*, *Plantago ovata*, and *Veronica agrestis*). In addition to these data, we also acquired WGS data for two specimens each from *Aloe vera* and *Linum usitatissimum* (flaxseed). These species were the third and fourth most common botanical ingredients found to co-occur with *Plantago* (plantain) based on a screen of twenty products marketed for digestive health on Amazon. No WGS data were available for the first and second most common co-occurring ingredients, *Rhamnus purshiana* (cascara sagrada) and *Cassia angustifolia* (senna). NCBI identification numbers for all companion data can be found in the appropriate Supplemental Tables (Tables S2, S5, and S7).

### 2.2. Nuclear Enrichment of Digitalis WGS Data

For one specimen from each of the five *Digitalis* species, we used *bbmerge.sh* and *bbduk.sh* from the BBTools suite (https://jgi.doe.gov/data-and-tools/bbtools/, Last accessed on 21 May 2021) to perform read merging and automated adapter removal. We used getOrganelle [39] to assemble circularized chloroplast genomes from these adapter-trimmed reads using kmer values of 21, 45, 65, 85, and 105 and a maximum of 50 extension rounds. Except for the two *Digitalis* specimens used in the multispecies mixes, all *Digitalis* read sets were screened to remove chloroplast-derived data. We first quality-trimmed raw *Digitalis* reads using *bbduk.sh* from the BBTools suite (qtrim = w, ktrim = r, trimq = 10, maq = 15, minlength = 50), then assessed quality with FastqQC [40]. After pooling the chloroplast assemblies generated above, we used *bbmap.sh* from the BBTools suite to map and screen these quality-trimmed reads against this multispecies chloroplast reference dataset, separating reads that mapped (presumably chloroplast-derived reads) from nuclear-derived reads.

### 2.3. Composite Genome Assembly

In order to identify nuclear loci that would be useful for *Digitalis* detection in the absence of a reference genome, we used the SISRS bioinformatics pipeline [37] to generate conserved, orthologous sequence data directly from raw sequencing reads. SISRS generates a so-called ‘composite genome’ (i.e., a *de novo* pan genome) by pooling reads proportionally across species to a final depth of ~10X genomic coverage, and then performing a single *de novo* genome assembly. Using a genome size estimate of 2 Gb for the genus [41], we used *reformat.sh* from the BBTools suite to subsample reads equivalently among *Digitalis* species and evenly among specimens therein, resulting in a final genome assembly dataset containing 20 Gb of primarily nuclear *Digitalis* data. We used Ray v.2.3.2-devel [42] to assemble the composite genome using the subsampled nuclear reads, default parameters, and a k-value of 31. This composite genome represents a ‘taxonomically-averaged’, common set of nuclear loci against which read data from all *Digitalis* species can be mapped and compared.

### 2.4. Nuclear Digitalis Read Mapping and SNP Dataset Development

SISRS uses read data from individual specimens, species datasets containing multiple individuals, or specimens pooled at a higher taxonomic level to profile allelic variation at each site in the composite genome. In this study, we profiled *Digitalis* data at the species level (i.e., specimen data pooled together by species) and at the genus level (i.e., pooling all nuclear *Digitalis* datasets together). In combination, these datasets allowed us to isolate (1) all nuclear sites that had a single, fixed allele that was present in all five *Digitalis* species (i.e., putative genus-informative DNA markers) as well as sites that were variable among the *Digitalis* species (i.e., putative species-informative single-nucleotide polymorphisms or SNPs).

### 2.5. Background Species Mapping and Data Filtration

For both species- and genus-level identification from multi-species mixtures, discernibility will be restricted if identifying alleles are also present in more distantly related taxa (i.e., if they are non-specific). To enrich our dataset for *Digitalis*-specific SNPs, we used SISRS to map WGS data from 34 non-*Digitalis* Plantaginaceae species onto the composite genome, along with one specimen of aloe and flax. We quality-trimmed all read sets using *bbduk.sh* from the BBTools suite as before, but, contrary to the *Digitalis* samples, we did not separate these datasets into nuclear and chloroplast fractions and mapped all quality-trimmed reads. Based on these mappings, we pruned both the genus- and species-level SNP datasets of sites that had read coverage from any of the non-*Digitalis* species.

The SNPs generated above represent sites with informative variation, pre-screened against close evolutionary relatives and other species known to co-occur in mixtures. These sites would represent a good starting dataset for confirmatory tests of single specimens; however, in mixed-species samples, that could contain any number of co-occurring species, sites can be further filtered down to reduce the impact of cross-species read mapping. Using the genus-level SNP set, consisting of sites with *Digitalis*-specific variation among Plantaginaceae, aloe, and flaxseed, we tallied the number of screened SNPs that occurred on each composite genome contig and calculated the proportion of SNPs relative to the total contig length. We then filtered both the genus- and species-level SNP sets down to just those deriving from contigs with at least the median number and proportion of genus-level SNPs. This filtering removes contigs (and their SNPs) that contain predominantly non-specific data and enriches the dataset for regions where *Digitalis* has experienced potentially informative mutations.

### 2.6. Generating Mixed Samples

**Figure 1:**
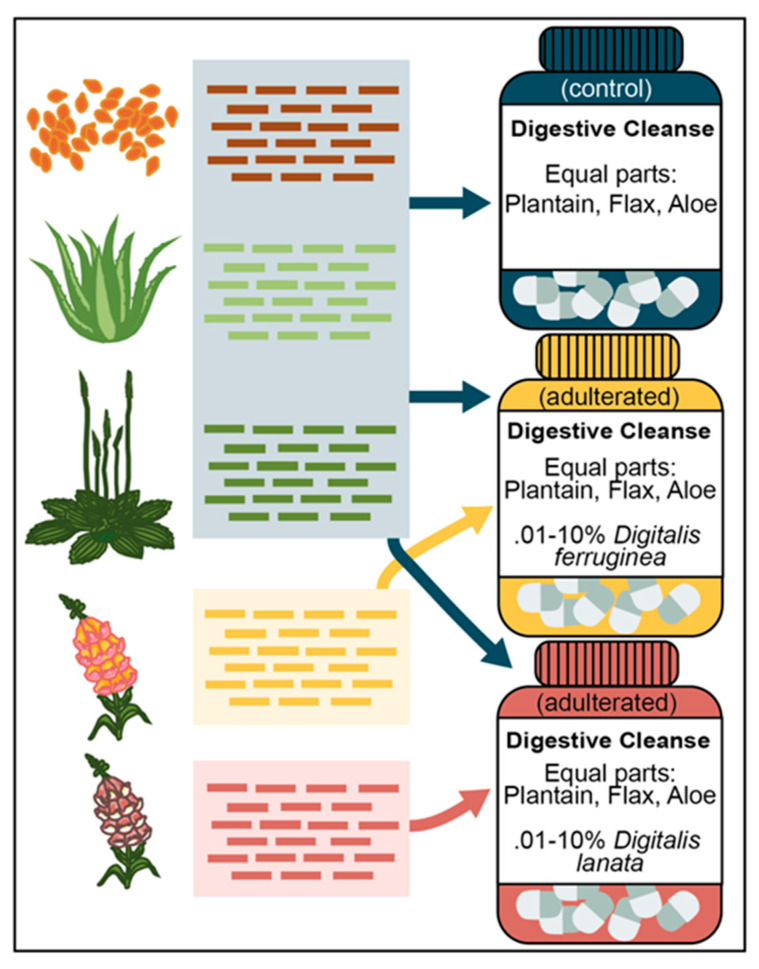
Overview of mock dietary supplement sample design. All mock supplement mixes were created in silico from sequencing data. The control sample consisted of equal parts *Plantago major*, *Aloe vera*, and *Linum usitatissimum* reads. The adulterated supplement mixes contained the same background as the control but also included 0.01%, 0.025%, 0.05%, 0.1%, 0.25%, 0.5%, 1%, 2.5%, 5%, and 10% of either *Digitalis lanata* or *Digitalis ferruginea* reads.

In order to assess the utility and sensitivity of the species- and genus-level SNPs for identifying *Digitalis* in multispecies mixtures, we spiked increasing amounts of WGS data from *Digitalis ferruginea* and *D. lanata* into a series of simulated mixtures containing equal amounts of WGS data from *Plantago major*, *Aloe vera*, and *Linum usitatissimum* (Figure 1). All read samples were quality-trimmed as before, and no chloroplast read separation was performed. For each mixture, we targeted a practically relevant base depth of ~1.88 Gb, equivalent to an Illumina MiSeq sequencing run with 8 multiplexed samples. For both *Digitalis* species, we generated ten sets of ‘adulterated’ data by adding *Digitalis* WGS data to final proportions ranging from 0.01% (187,500 bases) to 10% (187.5 Mb). Each mixture, including a control dataset that contained no *Digitalis* data, had ten pseudoreplicates generated by randomly subsampling reads from the quality-trimmed datasets using *reformat.sh* from the BBTools suite. For each pseudoreplicate, data from *D. ferruginea* and *D. lanata* were added to identical background mixtures (i.e., Replicate B1 was mapped twice using the same data for *Plantago, Aloe,* and *Linum*, while varying the *Digitalis* species), but background data varied among replicate sets (i.e., Replicates B1 and B2 contain different random subsets of *Plantago, Aloe,* and *Linum* data).

### 2.7. Screening Mixed Samples for Digitalis SNPs

We used SISRS to map each of the mix pseudoreplicates against the composite genome, and we surveyed the alleles present at each of the *Digitalis* genus- and species-informative sites that survived upstream screening and filtering. For each pseudoreplicate, we performed both a genus- and species-level inquiry; in both cases we queried the relevant SNPs and tallied (1) sites that had read coverage and (2) sites that contained the *Digitalis* genus- or species-informative allele.

#### 2.7.1. Genus-Level Digitalis Detection

For genus-level detection, we first used linear models in R [43] to assess the relationship between the number of *Digitalis* bases added to the mixture and the number of recovered *Digitalis* alleles. We then compared the number of *Digitalis* genus-informative alleles detected in the negative control group (where no *Digitalis* data were added) to the count from each pseudoreplicate group using *t*-tests in R, assessing significance at α = 0.05 (i.e., Does this set of pseudoreplicated samples contain more matching *Digitalis* genus SNPs than a sample with no *Digitalis* added?).

#### 2.7.2. Species-Level Digitalis Detection

For all species-informative SNP positions with coverage in each pseudoreplicate, we calculated the ratio of SNPs supporting and refuting each species assignment and assigned species based on the highest ratio (i.e., matching species-informative alleles/all species-informative SNP positions with coverage) and used a modified Z-score test [44] to statistically assess whether that SNP proportion was statistically distinct from the other species. As a median-based test, the modified Z-score test is robust when comparing small samples sizes (i.e., 5 species), and significance of all p-values was assessed after Bonferroni correction (α = 0.05/5 = 0.01). To reduce species assignments involving too few data, we only performed statistical species identification on pseudoreplicates where (1) there was read data in the mixture that covered 5 species-informative SNP positions for each *Digitalis* species, and (2) where the species with the highest ratio of matching species-informative SNPs was supported by at least 5 matching alleles.

## 3. Results

### 3.1. Assembly of the Nuclear Digitalis Composite Genome

Chloroplast genomes for each of the five survey *Digitalis* species were assembled with getOrganelle using between 662 Mb and 3.8 Gb of adapter-trimmed reads, and all assemblies resulted in a circular genome (Table S1). Trimmed base counts for the *Digitalis* samples ranged from 629 Mb to 24 Gb per species, and 90–98% of reads across datasets failed to map to the chloroplast dataset and were considered putatively nuclear (Table S2). Using 2 Gb as a genome size estimate for the genus, to achieve 10X genome coverage we targeted a subsampling depth of 4 Gb for each of the five species; however, the *D. purpurea* sample only had 572 Mb of nuclear data and we made up the difference equally among taxa. The composite genome assembly generated by Ray resulted in 1.8M largely fragmentary contigs totaling 334 Mb (N50: 666Kb; L50: 172; Table S3).

### 3.2. Nuclear Digitalis Mapping and SNP Dataset Generation

SISRS mapping of the nuclear *Digitalis* species datasets onto the composite genome resulted in base calls for 21 M–159 M sites per species (6.5–47.8% of sites; Table S4), while the *Digitalis* genus dataset containing data from all species resulted in base calls for 272 M sites (81.5% of sites; Table S4). Of these, 6.4 M sites had a single, fixed allele found in all five *Digitalis* species (Table S4). Among the five *Digitalis* species, species-informative SNP counts (sites with a fixed base for all species, and a unique allele for one species) ranged from 14 K–223 K sites per species (Table S4). After removing sites with coverage from non-*Digitalis* species (Table S5) we filtered contigs by the proportion and amount of *Digitalis*-specific sites, allowing SNPs from contigs where (1) at least 22.4% of sites in the contig were *Digitalis*-specific (median proportion) and (2) with at least 53 *Digitalis*-specific sites (median site count). After filtering, the final *Digitalis* genus SNP count was 2.4 M sites, while species-informative SNPs ranged from 6.3 K–101 K sites per species (Table S4).

### 3.3. Screening Mixed Samples

To create the simulated sample mixtures, we pooled equivalent amounts of trimmed WGS data from *Plantago, Aloe*, and *Linum* with increasing amounts of spike-in *D. ferruginea* or *D. lanata* data (Table S6) ranging from 0.01% to 10% (Table S7). Each simulated sample contained ~1.9 Gb of trimmed WGS data. We used SISRS to map all reads from each mix pseudoreplicate against the composite genome (n = 10 per spike species, per spike amount), and defined an SNP match as a site where either a genus- or species-informative allele was detected.

#### 3.3.1. Genus-Level Screening

**Figure 2:**
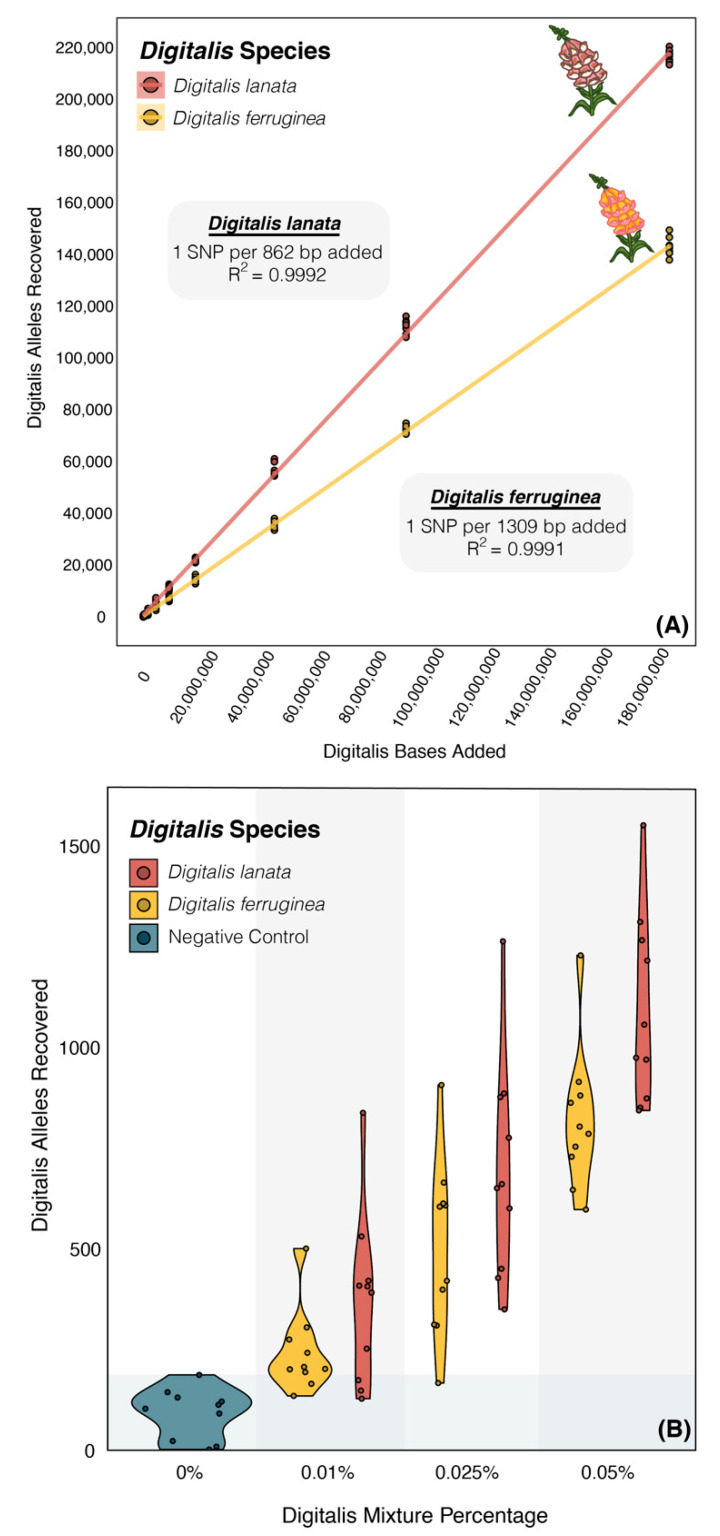
*Digitalis* detection using SISRS. (**A**) Spiking more WGS data from *Digitalis ferruginea* or *D. lanata* into simulated mixtures containing *Plantago, Aloe,* and *Linum* resulted in a strongly correlated increase in the number of *Digitalis* genus-informative alleles recovered from mixed samples (*p* < 2.0 × 10^−16^; R^2^ > 0.999). (**B**) At low concentrations of adulterating *Digitalis* (0.01–0.25%), the distribution of detected *Digitalis* genus-informative alleles overlapped with that of the negative control that had no *Digitalis* data added (grey bar), although group means were still significantly higher (all *p* < 2.5 × 10^−3^). When *Digitalis* data made up 0.05% or more of the data (~937 Kb), there was an unambiguously significant increase in detected alleles (all *p* < 3.47 × 10^−7^).

For mix pseudoreplicates spiked with *Digitalis* data, the number of recovered alleles indicating the presence of the genus *Digitalis* was highly correlated with the number of *Digitalis* bases added. *Digitalis ferruginea* resulted in ~1 new SNP match for every 1309 bases added (*p* < 2.2 × 10^−16^, R^2^ = 0.9991), and *D. lanata* resulted in 1 new SNP match for every 862 bases added (*p* < 2 × 10^−16^, R^2^ = 0.9992; Figure 2A; Table S8). The negative control samples with no *Digitalis* data added had positive hits for 8–193 sites (Figure 2B; Table S7), while spiking in the lowest amount of either *D. ferruginea* or *D. lanata* (~187 Kb or ~0.01%) resulted in a significantly higher average of 339 and 360 sites, respectively (both *p* < 2.5 × 10^−3^; Figure 2B; Tables S7 and S9). All *Digitalis*-spiked pseudoreplicate groups had a statistically higher average number of *Digitalis* genus matches relative to the negative control group (all *p* < 2.5 × 10^−3^; Table S9), but the distribution of matched sites overlapped with the negative control samples at the two lowest spike-in concentrations for both *Digitalis* species (0.01% and 0.025%, 187 Kb and 468 Kb) (Figure 2B).

#### 3.3.2. Species-Level Screening

**Figure 3:**
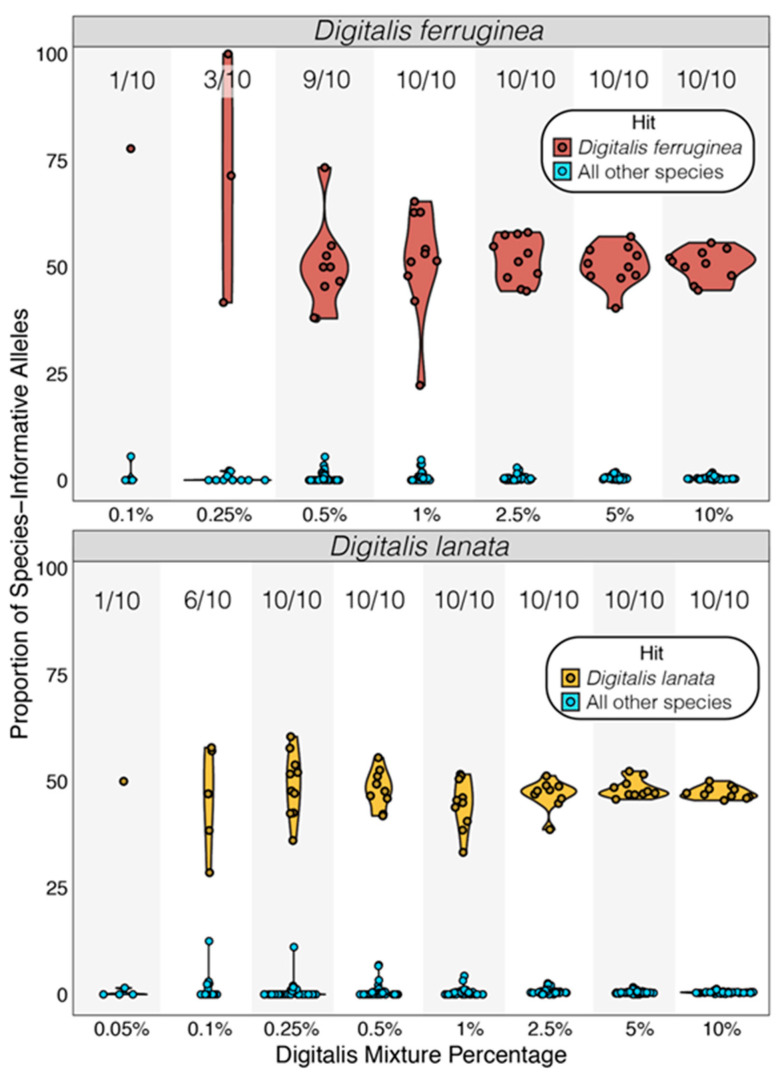
Accuracy of *Digitalis* species detection. For all mixture pseudoreplicates with sufficient data, the species of *Digitalis* was assigned based on the proportion of SNPs from each sample that contained species-informative alleles. In all cases the highest ratio of matching SNPs derived from the correct species, and all comparisons were significant based on modified Z-score analysis (all *p* ≤ 9.72 × 10^−35^). Proportions represent pseudoreplicates meeting the minimum data criteria. When *Digitalis* data accounted for 1% or more of the dataset (18.75 Mb), 10/10 pseudoreplicates could be identified, but limited data precluded robust species identification at some lower concentrations.

We queried the alleles present at all species-informative SNP positions and only classified samples where (1) each species had 5 SNP positions covered, and (2) at least 5 matching SNPs supported the top species hit. Out of the 100 *Digitalis*-spiked pseudoreplicates for each species (10 pseudoreplicates × 10 spike-in amounts), *D. ferruginea* had 53 pseudoreplicates that passed this filtering, while 67 *D. lanata* pseudoreplicates contained sufficient data. In each case, the adulterant species in each qualifying pseudoreplicate was correctly identified (Figure 3; all *p* < 9.7 × 10^−35^; Tables S10 and S11) and all pseudoreplicates could be classified when 18.75 Mb of *Digitalis* data (1% of the total data) was added (Figure 3; Tables S11 and S12). At lower *Digitalis* concentrations, some *D. ferruginea* could also be identified at 0.1% (n = 1/10), 0.25% (n = 3/10), and 0.5% (n = 9/10) (Figure 3; Tables S11 and S12), while *D. lanata* was correctly identified in all pseudoreplicates containing at least 4.6 Mb (0.25% of the total data; Tables S11 and S12) and also at 0.05% (n = 1/10) and 0.1% (n = 6/10) (Figure 3; Tables S11 and S12). In all cases, 44–77% of SNPs that associated with the spike-in species had the correct species allele, while the second-best match never reached above 5.6% (Figure 3; Table S12).

## 4. Discussion

Identifying ingredients of complex, mixed samples using DNA methods is an ongoing challenge, and the method that is chosen for a particular question must be carefully considered. Metabarcoding approaches often rely on well-curated reference databases, but due to the limited size of many of these datasets, care must be taken to choose marker regions that both (1) provide appropriate taxonomic discrimination while (2) also accounting for issues such as amplification errors, bias, and artifacts introduced by PCR. Additionally, metabarcoding approaches often preclude quantification beyond relative abundance, and relying on highly targeted techniques can also fail due to fragmented DNA [21], which is often found in highly processed food products and dietary supplements.

On the other end of the spectrum, whole-genome sequencing (WGS) approaches, including low-coverage genome skimming, circumvent many of the limitations associated with traditional metabarcoding or targeted locus methods. WGS data provides an untargeted snapshot of DNA in a sample; it bypasses the need for *a priori* marker selection, reduces negative biases associated with PCR amplification, and provides a more holistic, semiquantitative (with caveats), representation of sample composition [21]. Especially in the absence of a reference genome, analysis of WGS data typically relies on kmer binning [45,46,47], BLAST [48], or metagenomic assembly and classification of the resulting contigs using a database [49,50]. The availability of genome annotation data or useful marker sets in botanicals varies widely outside of model clades, and in many cases the taxonomic discrimination of analyses is severely limited by database robustness, or lack thereof. As an attempt to overcome these challenges, many WGS-based identification approaches still begin with a reduction down to smaller, more manageable data subsets, such as extraction of organellar reads [36,51], or specific sets of well-characterized genes [52]; yet, based on both their size (e.g., datasets containing few loci) and the nature of their heredity (e.g., in the case of maternal chloroplast inheritance), these data subsets also present challenges when identifying closely related species or hybrids.

Here we present a pipeline that relies only on WGS data, without the need for a reference genome, annotation data, or any external databases, to generate informative SNPs for the identification of toxic adulterants (*Digitalis*) in mixed botanical samples. This pipeline overcomes both the need for *a priori* marker selection and PCR amplification biases associated with metabarcoding, as well as the typical issues associated with work in non-model clades. While recent plastid-based analyses of *Anemopaegma* and *Veronica* yielded informative SNP counts in the thousands [53,54], here we identify over 2.4 million nuclear genus-informative SNPs for *Digitalis* that were screened against a background of *Plantago* (plantain)*, Aloe vera* (aloe)*,* and *Linum usitatissimum* (flax) (Table S4, Figure 1), as well as tens of thousands of species-informative SNPs. Rather than relying on a small subset of plastid reads or gene subsets, working from a large, untargeted nuclear dataset dramatically increased both our resolution and ability to recover orders of magnitude more SNPs when compared to many existing methods.

We simulated mixed samples based on practical data limits (~2 Gb per sample, equivalent to a MiSeq 600-cycle kit multiplexed eight ways) and at this depth unambiguous genus-level *Digitalis* detection was possible even in trace amounts when *Digitalis* data made up only 0.05% of the mixture (~1 Mb of spike-in data) (Figure 2B). Additionally, we see a highly-correlated increase in genus-level SNP recovery with respect to the amount of *Digitalis* data spiked into the mixture (Figure 2A), suggesting that this method is semiquantitative, supporting previous work [21]. Relative to the genus-informative SNP sets, there were fewer species-informative SNPs, and robust identification of species required around 10 times more data (0.5% of the mixture, or just under 10Mb) (Figure 3).

The nuclear loci generated by SISRS [37] were assembled using only *Digitalis* nuclear data, and as such, rescreening this dataset against species other than *Plantago*, *Aloe*, and *Linum* would theoretically allow for detection of *Digitalis* in mixtures containing any primary ingredients. Additionally, these datasets are dynamic, and more data from the existing *Digitalis* species or data from background species not included here could be analyzed without the need to generate a new composite genome from scratch. For instance, we screened our *Digitalis* markers against a single specimen of most outgroup taxa to reduce the amount of cross-species mapping; yet the unadulterated control samples had ~100 SNP matches that should have been *Digitalis*-specific (Figure 2B). In a real-world test case these sites too could be purged from the SNP lists for any downstream tests, and this iterative database refinement would serve to minimize false positives. Notably, the *Digitalis purpurea* sample used in this study resulted in an abnormally high number of species-specific SNPs relative to the other four *Digitalis* species screened (Table S4), which could be due to sample contamination, sequencing errors, or a high degree of evolutionary divergence in this species [55]. If this aberration is not biologically founded (i.e., if due to some systematic error), swapping that sample out for another and regenerating SNPs would also allow for the recovery of more SNPs.

While this pipeline is computational in nature, the SNPs identified in this study can just as easily be used for diagnostic primer design to develop targeted assays for the *Digitalis* genus, as well as specific species. This method does not rely on identifying markers within well-characterized loci and thus, is considerably less restrictive. Since the number of highly problematic toxic plants commonly found in foods and dietary supplements is finite, the development of additional composite genomes and SNP databases for taxa of concern is a feasible goal. In addition to NGS methods as employed here, these databases can be used to develop suites of targeted primer sets for use with common molecular methods such as PCR, or more specialized quantitative applications like qPCR or ddPCR. The pseudoreplicated, simulated adulteration study described here is largely intended as a proof of concept, yet also provided a clear roadmap for the application of SISRS for identifying species within mixed samples. Continued development of this method, including tests on real-world adulterated samples, may lead to the rapid expansion of both NGS-based and molecular tools for faster identification of toxic plants in foods and consumer products.

## 5. Conclusions

Here, we illustrate how shallow WGS data can be used to detect low concentrations of adulterants of a specific toxic or allergenic contaminant at the genus or species level with no pre-existing reference genome or annotation data. This provides a roadmap for the rapid generation of nuclear markers in non-model groups. Our results indicate that when provided with sufficient sequencing data of background materials, ~2 Gb of data can correctly identify adulteration with simulated contamination of as little as 0.05% of *Digitalis* to both the genus and species level in mixed botanical samples using a modified application of SISRS. In the 1997 case of *Digitalis* adulteration, we believe our method would have detected *D. lanata* in both the dietary supplement and the *Plantago* raw material, as well as provide semiquantitative information regarding the amount of *D. lanata* present.

## Data Availability

Raw data generated for this project are available under the GenBank BioProject PRJNA325670. Specific SRA accessions for each sample can be found in the Supplementary Tables S2 and S5.

## Supplementary Materials

The following are available online at https://www.mdpi.com/article/10.3390/foods10081794/s1, Table S1: Whole-genome sequence data used to assemble Digitalis chloroplast genomes using getOrganelle, Table S2: Digitalis whole-genome sequence data used in this study for composite genome assembly and SNP discovery, Table S3: SISRS assembly statistics for Digitalis composite genome, Table S4: Results of mapping Digitalis whole-genome sequencing data onto composite genome including filtering SNPs to include more Digitalis-specific contigs, Table S5: Non-Digitalis whole-genome sequence data used in this study for SNP masking, Table S6: Whole-genome sequence data used in this study for creating mixed pseudoreplicates, Table S7: Pseudoreplicated mix components and mapping statistics, Table S8: Results from linear regression of recovered SNPs versus Digitalis bases added, Table S9: Summary of genus-level classification results for mixed pseudoreplicates including total matching sites and proportion of sites that matched Digitalis, Table S10: Individual species-level classification data for each mix pseudoreplicate, Table S11: Species-level classification results for mix pseudoreplicates meeting or exceeding the minimum classification criteria, Table S12: Comparison of difference between average signal strength for top species match versus second highest species match.
